# Multipurpose prevention technologies for the prevention of unintended pregnancy, HIV, and other sexually transmitted infections: regulatory pathways and challenges

**DOI:** 10.3389/frph.2025.1591232

**Published:** 2025-05-13

**Authors:** Logan Donaldson, Robin Schaefer, Sarah Alhakimi, Ruth Akulu, Thesla Palanee-Phillips, Bethany Young Holt, Veronica Miller

**Affiliations:** ^1^ Forum for Collaborative Research, University of California, Berkeley, Washington, DC, United States; ^2^ School of Public Health, University of California, Berkeley, CA, United States; ^3^ Young Women’s HIV Prevention Council, Kampala, Uganda; ^4^ School of Public Health, Wits RHI, University of the Witwatersrand, Johannesburg, South Africa; ^5^ Department of Epidemiology, School of Public Health, University of Washington, Seattle, Washington, DC, United States; ^6^ Public Health Institute, Initiative for Multipurpose Prevention Technologies, CAMI Health, Sacramento, CA, United States

**Keywords:** multipurpose prevention technologies, HIV prevention, pre-exposure prophylaxis, contraception, regulation

## Abstract

Multipurpose prevention technologies (MPTs) are multi-indication products commonly focusing on the prevention of unintended pregnancy, HIV, and/or other sexually transmitted infections (STIs). MPTs have the potential to simplify product use and service delivery with reduced clinic visits, thus supporting improved product uptake, effective use, and cost-effectiveness. MPTs are complex products that typically include multiple active pharmaceutical ingredients (APIs), with two or more indications, and often use a device to deliver these APIs. These complexities create challenges when seeking regulatory approval. Products with previously approved APIs may be able to rely on bioequivalence (BE) studies, but still face challenges in formulation variation, drug-drug interaction, and fulfilling strict standards. MPTs that use new APIs and devices cannot rely on BE studies for approval and thus face further uncertainty, including clinical trial design for products with multiple indications and outcomes of interest. Efficacious standards of care for HIV prevention and contraception also necessitate active-control designs for registrational clinical trials, thus innovative trial designs may be needed. Compounding these challenges are special regulatory requirements for combination products, in addition to standards applied to individual API and device. Possible approval pathways for combination products exist within the US Food and Drug Administration and other global regulatory authorities, but their complexities and challenges are untested for MPTs. They are highlighted in this article to raise awareness around regulatory pathways for MPTs. In Sub-Saharan Africa, women of reproductive age are the largest percentage of new HIV infections. This, in combination with considerable rates of unintended pregnancy and rising sexually transmitted infection (STI) rates, highlights the need for products that address these complex sexual and reproductive health needs. Multipurpose prevention technologies (MPTs) commonly focus on the prevention of unintended pregnancy, HIV, and/or other STIs in one product. MPTs combine the use of multiple pharmaceutical drugs and often a medical device to address these interrelated challenges. This creates complications in the design of studies for MPTs and in understanding the process of approval from regulatory authorities. Regulatory authorities are responsible for ensuring the safety and effectiveness of health products, and in MPTs this is complicated by targeting the prevention of multiple indications, with differing study designs and challenges, in one product. There are mechanisms in place at some regulatory authorities to evaluate MPTs, but these pathways are untested by product developers and regulatory authorities alike. Some of these pathways are highlighted below. Collaboration between diverse stakeholders like regulators, academics, product developers, and community members is necessary to build consensus on the best steps to address these challenges. MPTs are a potential tool to successfully prevent interrelated sexual and reproductive health concerns, but regulatory challenges must be addressed for safe and effective products to reach those who need them most.

## Introduction

Worldwide, women continue to face interrelated sexual and reproductive health (SRH) challenges, including exposure to HIV, other sexually transmitted infections (STIs), and unintended pregnancies. High rates of HIV infections persist among women, particularly in the World Health Organization (WHO) African region, where, in 2023, women made up 62% of incident infections (1). There were an estimated 374 million new cases of the curable bacterial STIs chlamydia, gonorrhea, trichomoniasis, as well as syphilis globally in 2020 (2), and an estimated one in ten women were infected with human papillomavirus (HPV) (3), the key driver of cervical cancer. Further, in 2019, 29% of pregnancies in the African region were unintended, ranging from 10.8% in Nigeria to 54.5% in Namibia (4).

Innovation is essential to meet the complex preventive SRH needs and preferences of all adolescent girls and women (5). Multipurpose prevention technologies (MPTs) are products designed to simultaneously prevent unintended pregnancies, HIV and/or other STIs. Among those in development, the most common focus is on preventing unintended pregnancy and HIV. These products may be more acceptable than single-indication prevention products by offering more streamlined use and product delivery, reducing the number of healthcare visits necessary, and thus possibly improving uptake and effective use of HIV/STI prevention products and contraception (6). MPTs, such as the Dual Prevention Pill (DPP), a single co-formulated pill that combines oral tenofovir disoproxil fumarate and emtricitabine (TDF/FTC) as pre-exposure prophylaxis (PrEP) for HIV prevention and levonorgestrel/ethinylestradiol (LNG/EE) as a combined oral contraceptive (COC), have the potential to be a cost-effective tool to improve health outcomes (7). End-user research highlights preferences for MPTs over single-indication products and the importance of engaging end-users throughout the development process of MPTs to ensure that products will be used by those who could benefit from them (8).

Although condoms are the only currently available MPT for these indications, a range of products are at different stages of development. This includes pills, injections, implants, as well as several other non-systemic product forms such as vaginal rings, films, and gels (9). However, only the DPP is expected to reach markets in the next few years, pending regulatory approval, while most other MPTs are in early development stages. MPTs face challenges like coformulation of multiple drugs with different physiochemical properties, drug-drug interactions, and drug-device combinations (10). Critical challenges are the complex regulatory pathways and limited regulatory guidance on the requirements to secure regulatory approval. In this article, we discuss these challenges, complexities, and questions around approval processes and pathways and how they can be addressed, which is critical for MPTs to reach approvals efficiently.

## Complexities of MPTs

MPTs, by nature of their indications, warrant complex regulatory considerations. A single technology with multiple indications, often multiple active pharmaceutical ingredients (APIs), and frequently associated device considerations, creates a potentially challenging path for product approval. In the US, regulatory standards for MPTs are derived from the applicable requirements of the individual parts of each MPT, including drugs, biological products, and devices (11). MPTs are additionally considered a distinct category of combination medical products that special regulatory requirements may apply to (11). Standards of quality, safety, and efficacy apply to all parts of the MPT, just as they would to the individual products comprising the MPT (11). (5), The combination of evaluating the individual products, and the MPT as a whole, creates a unique challenge that has yet to fully be navigated in the MPT field.

There were at least 19 MPTs with an HIV prevention indication at varying stages of development, as of August 2024 (9). MPTs differ by approval status of included APIs, with some using previously approved APIs, others using unapproved APIs, and some using a combination of both. The diversity of MPTs in development ensures a single regulatory pathway to reach approval cannot be followed. Despite this, MPTs using only previously approved APIs with similar dosage, indication, and route of administration may face a less complex route to approval (11, 12), In the US, this can be done via an annotated new drug application (ANDA) based on established bioequivalence (BE) through pharmacokinetic (PK) studies [and pharmacodynamic (PD) studies, where applicable] (11). An example of this is the DPP, which may use this process at the US Food and Drug Administration (FDA), and is likely the closest MPT to market approval (13). Rather than a large, costly, and time-consuming phase-3 clinical trial to evaluate safety and efficacy, the DPP is compared to the PK of the reference products: TDF/FTC for HIV prevention and LNG/EE for contraception (13).

While some MPTs are being developed using previously approved APIs, there are challenges that may arise in BE studies that can prevent or delay product approval. These have been seen in single indication fixed-dose combination (FDC) products (14), and will be present in multi-indication combination products for which BE is generally more difficult to demonstrate than for a single API (12). Formulation complexities in FDC products, like differing release profiles, introduce challenges in PK BE (14). Further, in combination products (single or multi-indication), drug-drug interactions must be understood from both PK and PD perspectives to ensure there is no antagonistic effect of the interaction (14). This may be of particular concern where some antiretroviral drugs can affect the metabolic uptake of hormonal contraception, thus impacting the efficacy of the product (12). For MPT formulations that use new APIs, drug-drug interaction must also be considered, but the pharmaco-profiles of these products may be unknown or understudied.

Using BE studies may be the most direct route to approval for some MPTs, but questions arise when considering an MPT that uses both approved and unapproved APIs. It is unknown if BE studies can be used to evaluate previously approved API(s) co-currently, in a single study, along-side a clinical trial for the previously unapproved API(s), or if this must be demonstrated in separate studies, prior to a study for the MPT as a whole. Moreover, the ability to use BE studies for MPTs using a differing delivery method or route than the API was initially approved for, is undetermined.

Further complicating the regulatory approval of these products is the risk–benefit analysis for MPTs. In the US FDA's “therapeutic context”, the risk associated with the use of the product must be outweighed by the demonstrated benefits, but with higher safety standards when evaluating preventative medications (15). For example, there are numerous multi-indication cancer treatment drugs approved for patients not responding to available treatment (16). These likely had higher risk tolerance in regulatory evaluation than MPTs with HIV prevention indications, due to the condition of the target population (15). In HIV pharmaceutical development, this therapeutic context may contribute to treatment approval frequently being sought for antiretroviral drugs before pursuing approval for a prevention indication. Finally, multiple MPTs aim to use long-acting and/or extended-release products that are expected to be used in healthy individuals for long periods of time, complicating risk assessments (17). However, multi-indication vaccines such as the Measles, Mumps, and Rubella vaccine (12) demonstrate that multi-indication prevention products can reach approval despite lower risk tolerance for prevention products.

## Questions on clinical trial designs for MPTs

Where BE is insufficient for regulatory approval, clinical trial data to demonstrate safety and efficacy are needed. Standards of clinical study design for HIV PrEP products and contraception differ. Trials for HIV PrEP have previously utilized a randomized placebo-controlled design, but, due to increasing availability of safe and efficacious HIV prevention options, these are no longer considered ethical (18). Contraception studies often use the Pearl Index design, which represents the number of contraceptive failures per 100 person-years of exposure (5). (19), Limitations in the Pearl index have been identified, including decreasing contraceptive failures with increased clinical trial time due to the likelihood of pregnancy decreasing overtime (19). Thus, life table analyses, which give the contraceptive failure rates for each month of use, providing cumulative failure rates, are required in addition to the Pearl Index by both the European Medicines Agency (EMA) and US FDA in contraceptive clinical trials (19). Therefore, it is likely that a superiority or non-inferiority trial design with an active control arm will need to be used in combination with the Pearl Index/Life Table design for MPTs (5, 10), Whether this can be done for an MPT evaluating HIV prevention and contraception in a single trial, or must be done in multiple trials, is yet to be seen. Separate considerations must be made for products with indications for the prevention of STIs such as HPV, chlamydia, or gonorrhea.

The need for active-control trial designs presents challenges as MPTs progress to later-phase studies. Superiority and non-inferiority trials compare novel agents to an active control (10, 20), often the standard of care (SOC), although what constitutes the SOC in HIV prevention and contraception varies over time and between countries. For HIV prevention, there are multiple efficacious PrEP options, including long-acting injectable cabotegravir (CAB-LA), which has been found to reduce the risk of HIV acquisition by 79% compared to TDF-based oral PrEP in two large clinical trials (21). In reversable contraception, there are two intrauterine methods that display a failure rate of under 1% and six hormonal methods that have failure rates between under 1%–7% (22). The presence of highly-effective active-control groups requires trials to have large sample sizes to adequately power studies, both increasing study cost and time (10, 20).

In HIV prevention studies, it has been proposed to use a design of comparing a counterfactual estimate of the HIV incidence among people not on PrEP to the incidence among those on the study drug (18, 20). This trial design, also known as the counterfactual placebo, uses an external control group that can be derived using several approaches, such as a recent infection testing algorithm (RITAs) via HIV recent infection assays (18). This trial design has been implemented to evaluate the efficacy of a six-monthly injection with lenacapavir for PrEP in the PURPOSE 1 and PURPOSE 2 trials (23), but it is untested in the MPT field.

## Existing regulatory approval pathways

Compounding complexities around MPTs and study design questions are ambiguous and further obfuscate complex approval paths that regulatory authorities currently have for MPTs. To date, most research on the development of MPTs was conducted by academic groups or small companies supported largely by the U.S. government via the US Agency for International Development (USAID) or National Institutes of Health (NIH) (17), while the majority target population for these products are women of reproductive age in the African region (24). With the majority of MPTs being developed by US based organizations, many MPTs may seek approval from both the US FDA and African regulatory authorities.

The US FDA has an Office of Combination Products (OCP) but the organization's guidance titled “Principles of Premarket Pathways for Combination Products” notes that the combination product will be assigned to a single lead center that has primary jurisdiction for that product's regulation (11). For MPTs, this could be the Center for Drug Evaluation and Research (CDER), Center for Biologics Evaluation and Research (CBER), or Center for Devices and Radiological Health (CDRH) within the US FDA. The lead center is based on the product's primary mode of action (PMOA) and will work with other centers within the FDA to ensure appropriate regulation, but a request to change the lead center can be submitted via a request for designation (RFD). A pre-RFD can be obtained to receive informal classification feedback prior to a formal RFD, but the OCP has final say on product classification. The lead center is the product sponsor's primary contact point at the FDA, and all communication should be through this center regardless of the information that is being requested on the product. The guidance states that combination products that seek separate marketing authorization for each constituent part, such as a new drug application (NDA) for a drug and a premarket notification for a device, create “distinct” considerations (11). Further, the guidance allows sponsors to discuss with the different centers and OCP how to ensure efficient engagement during review of submissions and ensures that appropriate center representation will be present to conduct reviews in a timely manner. The marketing application type submitted coincides with the PMOA, but each constituent part of the combination product should be evaluated as if they were going to be reviewed via separate applications, thus collecting the appropriate information to ensure quality, safety, and efficacy of each constituent part. Early and consistent interaction with the FDA via meetings, such as pre-investigational new drug meetings, is key to meeting product requirements. In addition to this guidance, developers must consider the International Conference on Harmonization guidelines and Chemistry Manufacturing Controls, when generating evidence about their MPTs. Despite existing guidance, there is limited experience in the MPT field of these cross-center applications and reviews, and particularly smaller organizations may struggle with the intricacies involved.

The probable desire for product approval in the United States and other countries, notably in the African region, creates further complications, both for product developers that may have limited experience in international and for regulatory authorities that may have limited resources and technical capacity to evaluate such complex products (25). This may lead to lengthy registration times for these products in countries where there is the highest need. Collaborative approaches based on the concept of harmonization and reliance, in which regulatory authorities utilize the work completed by another authority, have the potential to lead to more efficient product registration. After completing a pilot in 2012, the World Health Organization (WHO) launched a pilot collaborative registration procedure (CRP) in which product assessment dossiers on WHO-prequalified products are shared with participating countries (26), promoting more efficient regulatory approval processes. The WHO prequalification program assesses finished pharmaceutical products and APIs related to specific therapeutic areas, including HIV and reproductive health, and ensures quality, safety, and efficacy of medical products. The WHO CRP has led to accelerated registration of WHO-prequalified products in low- and middle-income countries (LMICs) (27, 28), The procedure has since been expanded to facilitate the sharing of assessment reports of products reviewed by a stringent regulatory authority (SRA), although the US FDA does not currently participate in this process (25).

WHO has prequalified products and ingredients utilized in the development of MPTs, such as the dapivirine vaginal ring for HIV prevention and levonorgestrel-based tablets and devices for contraception. WHO issues invitations for expressions of interest for evaluation for prequalification based on need identified by a WHO disease program. This is typically done when the product is included in the WHO List of Essential Medicines or a WHO guideline. After a WHO recommendation is issued, the most expedited route to prequalification is the alternative listing procedure, but this only covers products that received a positive opinion by the EMA under the EU-Medicines for all (EU-M4all) procedure, tentative approval by the US FDA, or approval by the Australian Therapeutic Goods Administration. However, the alternative listing procedure may not be utilized given that many MPTs under development are likely to seek full US FDA approval and manufacturers may have limited capacity to simultaneously apply to another SRA. Additionally, while WHO guidance exists for the prequalification of FDC products (29), there are no specific considerations for products with multiple indications. Therefore, prequalification of MPTs may be feasible through existing pathways but lack of experience with prequalifying MPTs may cause additional delays.

## End-user and community perspectives

Regardless of regulatory or product development challenges, end-user and patient engagement in the development process is critical to ensuring the manufacture of innovative products that best serve those who will use them (30). End-users as active participants in each step of the development process, from pre-clinical through post-marketing, not only facilitate the development of products that address the health needs of the target populations (31), but also potentially increases the efficiency of development and product uptake (30). End-user and patient participation has recently been encouraged in drug development by the US FDA's Patient Focused Drug Development (PFDD) guidance and the European Patients’ Academy on Therapeutic Innovation (EUPATI) (31). End-user perspective research is taking place as MPTs are being developed. One example is the Quatro study, which evaluated the acceptability of four distinct vaginally inserted HIV prevention products, that may eventually be adapted into MPTs (32). This type of work must continue throughout the development process to ensure safe and effective MPTs are accepted and understood by end-users.

## Conclusions and next steps

The nature of MPTs creates unique considerations for regulatory approval processes. Each step of study development and regulatory approval is complicated by issues like API approval status, drug-drug interactions, device-drug combinations, and high standards for preventive products. Difficult questions regarding the study of MPTs, especially in products that cannot rely solely on BE for approval, are created by these complexities. Where clinical trials are required, the design of studies are unclear. Highly efficacious PrEP and contraceptive standards of care necessitate active-control study designs, which likely require large sample sizes to be sufficiently powered to establish non-inferiority or superiority, limiting the feasibility of such studies. Therefore, innovative trial designs may be needed, such as the use of an external control group to measure the impact of HIV prevention products. The FDA provides combination product guidance, but the existing cross-center approach is complex and creates uncertainties for developers. To reach appropriate end-user populations, approval of products in LMICs will be necessary. WHO prequalification may offer an efficient, alternative, pathway for regulation by reliance; however, there is a lack of experience with prequalification of MPTs, the US FDA does not participate in the WHO CRP, and products fully approved by the agency are not eligible for the alternative listing procedure for WHO prequalification. Regulatory harmonization and the establishment of the African Medicines Agency (AMA), as a specialized agency of the African Union, have the potential to improve efficiency in regulatory approval in Africa (33); however, these efforts are ongoing and pathways are yet to be established.

Multistakeholder collaboration, including academics, community members, product developers, and regulators, among others, can help foster consensus on regulatory pathways. Such a collaborative process has been used in establishing innovative HIV PrEP clinical trial designs (18), and could be used to clarify essential questions for MPTs. Considering recent changes to the global health landscape under the current US administration, threatening SRH treatment and prevention research and programs (34), collaboration is needed more than ever to ensure safe and effective products are reaching those who need them most.

## Data Availability

The original contributions presented in the study are included in the article/Supplementary Material, further inquiries can be directed to the corresponding author.
